# Classical HLA class II associations with ALS in Kuwait reveal a DR7–DQ2.2 risk haplotype

**DOI:** 10.3389/fimmu.2026.1820694

**Published:** 2026-05-08

**Authors:** Mohammed Dashti, Faye AlAbdulghafour, Ohood AlMutairi, Anwar Mohammad, Md Zubbair Malik, Khadeja AlRefaie, Rasheeba Nizam, Sindhu Jacob, Ammar Chalabi, Ahmad Al khleifat, Fahd Al-Mulla

**Affiliations:** 1 Translational Research Department, Dasman Diabetes Institute, Kuwait City, Kuwait; 2 Department of Neurology, Kuwait Institute of Medical Specializations, Kuwait Ministry of Health, Kuwait City, Kuwait; 3 Maurice Wohl Clinical Neuroscience Institute, Institute of Psychiatry, Psychology and Neuroscience, King’s College London, London, United Kingdom; 4 King’s College London, Institute of Psychiatry, Psychology and Neuroscience, London, United Kingdom; 5 King’s College Hospital, NHS Foundation Trust, London, United Kingdom

**Keywords:** amyotrophic lateral sclerosis, antigen presentation, haplotype, HLA typing, neuroinflammation

## Abstract

**Background:**

Genome-wide association studies have implicated the human leukocyte antigen/major histocompatibility complex (HLA/MHC) region in amyotrophic lateral sclerosis (ALS) susceptibility, and immune dysregulation is increasingly recognised as a modifier of disease course. High-resolution HLA data for ALS remain confined to European and East Asian ancestries. We tested whether classical HLA class I and class II variation contributes to ALS susceptibility in a Kuwaiti cohort.

**Methods:**

We analysed 38 unrelated ALS cases (mean age, 57.4 years; 63.2% male) and 150 population-matched controls (mean age, 57.0 years) from Kuwait. HLA-A, -B, -C, -DRB1, -DQA1, and -DQB1 alleles were typed at two-field resolution using HLA-HD from next-generation sequencing (NGS) data. Allele, haplotype, and amino-acid residue associations were tested in BIGDAWG with locus-specific Bonferroni correction, with additional across-locus sensitivity analysis. Significant class II residues were mapped onto AlphaFold 3 structural models and interpreted alongside published class II crystal structures.

**Results:**

No class I allele, haplotype, or residue remained significant after correction. In class II, DQA1*02:01 [odds ratio (OR) = 3.18, 95% confidence interval (CI) 1.67 to 5.95, *p*_c_ = 0.0007], DRB1*07:01 (OR = 3.00, 95% CI: 1.58–5.59, *p*_c_ = 0.001), and DQB1*02:02 (OR = 2.48, 95% CI: 1.28–4.69, *p*_c_ = 0.03) were enriched in cases. The extended haplotype DQA1*02:01~DQB1*02:02~DRB1*07:01 (DR7–DQ2.2) conferred increased odds of ALS (OR = 3.11, 95% CI: 1.53–6.19, *p*_c_ = 0.002). Amino-acid residue analysis identified convergent risk positions in DQα1 (positions 47, 52, and 54; OR = 3.18, *p*_c_ = 0.006) and DRβ1 (positions 11, 13, 14, 25, and 30; OR = 2.84, *p*_c_ = 0.03) that map to the peptide-binding groove and correspond to the defining motifs of DQA1*02:01 and DRB1*07:01.

**Conclusion:**

Using high-resolution NGS-based HLA typing in a Kuwaiti cohort, we identified a class II risk signal for ALS centred on the DR7–DQ2.2 haplotype with convergent residue-level support in the peptide-binding domains. These findings support a contribution of class II-restricted antigen presentation to ALS susceptibility and warrant functional validation and replication in larger, independent Middle Eastern cohorts.

## Introduction

Amyotrophic lateral sclerosis (ALS) is a progressive neurodegenerative disease characterised by selective loss of upper and lower motor neurons in the brain and spinal cord. Patients typically develop muscle weakness and atrophy with fasciculations, cramps, and spasticity, progressing to dysarthria, dysphagia, and, ultimately, respiratory failure, which is the leading cause of death within a median of 5 years from symptom onset. ALS is clinically and genetically heterogeneous and is widely regarded as a complex, multifactorial disease shaped by genetic predisposition, environmental exposures, and their interactions (1–3).

ALS is relatively rare, with a global median incidence of 1.68 per 100,000 person-years and a median point prevalence of 5.20 per 100,000, though substantial geographic variation exists (4). Epidemiological data from the Middle East, including Kuwait, remain sparse. Gulf War veteran cohorts have shown elevated prevalence (up to ~19.7 per 100,000), a difference often attributed to conflict-related environmental exposures (5). Still, population-based genetic studies in this region are largely absent.

More than 50 genes have been implicated in ALS susceptibility, with pathogenic variants in *C9orf72*, *SOD1*, *TARDBP*, and *FUS* accounting for the majority of explained familial cases and a fraction of sporadic disease (6–11). GWAS have additionally identified common variants of smaller effect in genes related to neuronal homeostasis, vesicle trafficking, RNA metabolism, and immune pathways (12–14). Twin-based heritability is estimated at approximately 60% (15), yet a significant proportion of both sporadic and familial cases remains without a molecular diagnosis (16), pointing to unexplored genetic mechanisms.

Among these, immune-related pathways have attracted increasing attention. ALS is not an autoimmune disease, yet immune dysregulation, particularly in antigen presentation, modifies disease susceptibility, progression, and survival (17). Neuropathological studies consistently show microglial activation in the motor cortex and spinal cord of patients with ALS, with expression of human leukocyte antigen (HLA)-DR, a class II major histocompatibility complex (MHC) antigen-presenting molecule. Concurrent upregulation of the kynurenine pathway points to an inflammatory and excitotoxic environment that likely accelerates motor neuron loss (18–21). ALS-causal genes reinforce this connection: both *C9orf72* and *FUS* downregulate core MHC-II components (*HLA-DRA*, *HLA-DRB1*, and *CD74*) in haematopoietic progenitors and microglia, and loss of *C9orf72* function impairs innate and adaptive immunity more broadly (3, 22–24). Large GWASs identify immune-related risk loci, including variants in the HLA region and near *TNIP1*/*GPX3*, with pathway enrichment analyses implicating immune signalling (12, 25–27). In patient cohorts, elevated circulating cytokines and altered T-cell and natural killer (NK)-cell profiles correlate with faster progression, and early trials of tocilizumab, an interleukin-6 (IL-6) receptor antagonist, have provided preliminary evidence that targeting these alterations can slow decline (28).

Classical HLA class I and II genes are central to the antigen-presenting machinery, and several studies have examined whether specific HLA alleles contribute to ALS susceptibility. A systematic review of earlier candidate-gene studies from the United States, Scotland, Guam, Israel, Denmark, and England identified HLA-A9 and HLA-DR4 as potentially protective and HLA-B35 as a possible risk allele. However, the effect sizes were modest, and replication with modern high-resolution typing was recommended (29). In large European-ancestry cohorts, GWASs have implicated the broader HLA/MHC locus in ALS, with rs9275477 as the lead variant. Still, the association has not been resolved to a specific classical allele or haplotype. Cross-trait analyses also show colocalisation at this locus between ALS and Alzheimer’s disease (12, 30). An ecological analysis across 14 Continental Western European countries found that 59.8% of surveyed HLA alleles correlated negatively with motor neuron disease prevalence, with several class I alleles (HLA-A*26:01, A*32:01, and C*12:03) showing the strongest inverse associations; a smaller group, including HLA-B*07:02, B*15:01, B*40:01, and C*03:03, correlated positively (31). Outside European populations, the HLA-DRA/DRB5 region has been associated with ALS in Han Chinese individuals, where the rs9268856 AA genotype conferred both increased risk and shorter survival, supporting a class II contribution to disease course (32).

Despite these advances, HLA associations with ALS have been under-explored for decades, with virtually all modern high-resolution data confined to European and East Asian ancestries. Middle Eastern populations, which carry distinct HLA allele and haplotype frequencies shaped by different demographic histories, have not been examined. Accordingly, the present study derives classical class I and class II HLA alleles, haplotypes, and amino-acid residues from next-generation sequencing (NGS) data in Kuwaiti ALS cases and population-matched controls to assess HLA associations with ALS in this population. To ensure accurate typing, we used HLA-HD (33), selected based on our prior benchmarking of NGS-based HLA typing tools against clinical-grade methods, which ranked it highly for performance, efficiency, and reliability on whole-exome sequencing (WES) data (34).

## Materials and methods

### Ethics statements and study cohort

This case–control study included 38 unrelated patients with ALS recruited from neurology clinics across Kuwait and 150 unrelated adult population-matched controls from a previously published Kuwaiti exome cohort (35). ALS was diagnosed by a consultant neurologist according to the revised El Escorial criteria (36), which define Clinically Definite, Clinically Probable, Clinically Probable laboratory-supported, and Clinically Possible ALS based on the pattern of upper and lower motor neuron signs across bulbar, cervical, thoracic, and lumbosacral regions, supported by electrophysiological investigation and exclusion of alternative diagnoses. Population of origin was determined from the nationality field recorded on the Kuwaiti civil ID, the standard legally issued identifier documenting national origin in Kuwait. Cases and controls were matched on this variable. The cohort consisted predominantly of Kuwaiti nationals, with a minority of cases holding other Arab nationalities (**Table 1**). Age at symptom onset was defined as the age in completed years at which the patient or a close informant first noted focal motor symptoms attributable to ALS (focal weakness, cramps, fasciculations, dysarthria, or dysphagia), as documented at the first neurology clinic assessment. This definition follows the standard convention in ALS epidemiological and clinical research. ALS case recruitment and whole-genome sequencing (WGS) were approved by the Kuwait Ministry of Health Ethics Committee. The Dasman Diabetes Institute Ethical Review Committee approved the use of the control cohort. The study was conducted in accordance with the Declaration of Helsinki, and all participants provided written informed consent.

**Table 1:** Clinical and demographic characteristics of study participants.

Characteristic	Patients with ALS (*n* = 38)	Healthy controls (*n* = 150)
Age, mean (years)	57.4	57
Sex, *n* (%)		
Male	24 (63.2)	50 (33.3)
Female	14 (36.8)	100 (66.7)
ALS site of onset, *n* (%)		—
Spinal	25 (65.8)	—
Bulbar	8 (21.1)	—
Flail leg	1 (2.6)	—
Flail arm	2 (5.3)	—
Other/Unspecified	2 (5.3)	—
ALS clinical phenotype, *n* (%)		—
Classical ALS	24 (63.2)	—
Bulbar phenotype	3 (7.9)	—
Upper motor-neuron predominant	2 (5.3)	—
Flail legs	1 (2.6)	—
Flail arms	2 (5.3)	—
Classical ALS (bulbar onset)	6 (15.8)	—

### Sequencing

Whole-genome libraries for ALS cases were prepared using the Illumina DNA Prep (M) Tagmentation kit and sequenced on an Illumina NovaSeq 6000; full details of recruitment, clinical characterisation, and sequencing are provided in AlAbdulghafour et al. (in preparation). Control exomes were captured with the Nextera Rapid Capture Exome kit (Illumina) and sequenced on an Illumina HiSeq 2500 (35). Raw paired-end FASTQ files from both cohorts served as input for HLA typing.

### HLA typing

Classical HLA class I (A, B, and C) and class II (DRB1, DQA1, and DQB1) genotypes were inferred at two-field resolution using HLA-HD v1.7.0 (33) against IPD-IMGT/HLA reference v3.56 (37). HLA-HD was selected on the basis of its high performance in our benchmarking of NGS-based typing tools on whole-exome data (34). Platform heterogeneity (WGS in cases and WES in controls) was mitigated by harmonising all calls at two-field resolution, where cross-platform concordance exceeded 95% across 29 HLA loci in our prior evaluation (34).

### Statistical tests

Allele, haplotype, and amino-acid residue associations with ALS were evaluated using BIGDAWG v3.0.3 (38) in R v4.0 at two-field resolution, with non-strict binning, allele trimming enabled, and zero missing calls permitted. Association tests were conducted in three parallel BIGDAWG runs reflecting the hypothesis structure: (i) the three classical class I loci (HLA-A, -B, and -C); (ii) the three classical class II loci (HLA-DRB1, -DQA1, and -DQB1); and (iii) an extended eight-locus run (HLA-A, -B, -C, -DPA1, -DPB1, -DRB1, -DQA1, and -DQB1) used solely for the extended haplotype analysis. Allele frequencies were calculated on an allele-count basis, with each individual contributing two alleles per locus; the frequency of a given allele was therefore defined as the number of observed copies divided by 2*N*, where *N* is the number of successfully genotyped individuals at that locus. For each allele, case–control comparisons were performed using a 2 × 2 contingency table of allele counts, comparing the target allele with all remaining alleles at the same locus. BIGDAWG estimated allele and haplotype frequencies by expectation-maximisation, computed odds ratios (ORs) with 95% confidence intervals (CIs) from case–control contingency tables, and applied Fisher’s exact test when the expected count in any cell of the 2 × 2 contingency table was small (<5), and the chi-squared test otherwise. Hardy–Weinberg equilibrium was assessed in controls for quality screening.

Low-frequency alleles and haplotypes were pooled into a “binned” category and excluded from single-marker interpretation. For amino-acid residues, omnibus tests were performed across all states at each polymorphic position, followed by state-specific contrasts where the omnibus test was significant.

All tests were two-sided. Nominal significance was set at *p* < 0.05. Multiple testing was corrected by the Bonferroni method within each locus or haplotype/residue set as the primary analysis, and across all non-binned allele-level tests across the six classical loci combined (*n* = 41) as a sensitivity analysis. Corrected *p*-values (*p*_c_) < 0.05 were considered significant. Primary analyses were conducted without adjusting for sex, consistent with standard approaches for autosomal HLA genes, which lack established sex-linked expression biases.

To assess whether the associated class II alleles represented independent effects or a shared haplotypic signal after accounting for linkage disequilibrium across the DR7–DQ2.2 block, we performed stepwise logistic regression using carrier status (0/1) for DQA1*02:01, DRB1*07:01, and DQB1*02:02. Univariate, pairwise, and three-allele joint models were fitted in R using the glm function with a binomial family specification. For comparison, we also fitted a single-predictor model using DR7–DQ2.2 haplotype carrier status. The three-allele joint model and the single-haplotype model were compared by the likelihood-ratio test.

### Genotype–phenotype analysis

To explore whether the significant class II alleles influenced clinical presentation, we examined age of symptom onset (available for 28 patients), site of onset, sex, and respiratory end point status amongst the 38 ALS cases. End points were defined as tracheostomy or non-invasive ventilation (NIV) >23 h/day; patients without an event were censored at the last follow-up (2025).

Age of onset was modelled by linear regression adjusting for sex and site of onset, with carrier status for each significant allele or the composite risk haplotype as the independent variable. Group differences were also assessed by Mann–Whitney *U* tests.

Time to the respiratory end point was evaluated by Cox proportional hazards regression. Given only six events amongst 12 patients with documented end point data, models were restricted to univariate and minimally adjusted analyses (39). Kaplan–Meier curves were compared by the log-rank test. All genotype–phenotype analyses and plots were generated in R v4.5.0.

### Structural modelling

Protein sequences for HLA-DQA1*02:01 and HLA-DRB1*07:01 were retrieved from the IPD-IMGT/HLA database (Release 3.62, 2025-10). Individual chain structures (DQA1 alpha-chain and DRB1 beta-chain) were predicted using AlphaFold 3 (40) and visualised in PyMOL (41), highlighting the polymorphic residues identified in the association analysis. These models depict individual chains rather than full heterodimers; groove architecture should be interpreted in light of known crystal structures of intact class II complexes (42, 43).

## Results

### Clinical characteristics

Thirty-eight ALS cases and 150 controls were analysed (**Table 1**). Mean age was similar between groups (57.4 vs. 57.0 years). Cases had a higher proportion of men (63.2% vs. 33.3%), consistent with the known male predominance in ALS. Most cases were Kuwaiti citizens (86.8%); five were nationals of neighbouring Arab countries. Spinal onset predominated (65.8%), followed by bulbar onset (21.1%). Classical ALS was the most common phenotype (63.2%).

### HLA allele associations

No class I allele (HLA-A, -B, or -C) reached significance after Bonferroni correction (**Supplementary Tables 1**-**S3**).

Three class II alleles surpassed locus-specific Bonferroni thresholds (**Tables 2**–**4**): DQA1*02:01 (OR = 3.18, 95% CI: 1.67–5.95, *p*_c_ = 0.0007), DQB1*02:02 (OR = 2.48, 95% CI: 1.28–4.69, *p*_c_ = 0.03), and DRB1*07:01 (OR = 3.00, 95% CI: 1.58–5.59, *p*_c_ = 0.001). Several additional alleles reached nominal significance but did not survive correction. Pooled low-frequency (“binned”) categories are not interpreted individually.

**Table 2:** HLA-DQA1 allele frequencies and association with ALS.

Locus	Allele	Controls frequency	ALS frequency	OR (95% CI)	*p*-value	*p*_c_*
DQA1	01:01	0.05	0.04	0.84 (0.15–3.12)	0.79	1
DQA1	01:02	0.16	0.21	1.4 (0.69–2.72)	0.30	1
DQA1	01:03	0.11	0.03	0.23 (0.03–0.93)	0.03	0.29
DQA1	01:05	0.04	0.01	0.29 (0.01–2.02)	0.21	1
DQA1	02:01	0.13	0.32	3.18 (1.67–5.95)	0.00007	0.0007
DQA1	03:01	0.09	0.11	1.24 (0.46–2.98)	0.61	1
DQA1	03:03	0.06	0.05	0.87 (0.21–2.76)	0.81	1
DQA1	05:01	0.18	0.14	0.79 (0.35–1.64)	0.51	1
DQA1	05:05	0.14	0.05	0.35 (0.09–1.02)	0.04	0.44
DQA1	Binned			0.68 (0.13–2.46)	0.55	1

*p*_c_* = Bonferroni-corrected *p* within DQA1. “Binned” pools low-frequency alleles; not interpreted individually.

**Table 3:** HLA-DQB1 allele frequencies and association with ALS.

Locus	Allele	Controls frequency	ALS frequency	OR (95% CI)	*p*-value	*p*_c_*
DQB1	02:01	0.17	0.14	0.83 (0.37–1.72)	0.60	1
DQB1	02:02	0.13	0.28	2.48 (1.28–4.69)	0.003	0.03
DQB1	03:01	0.12	0.05	0.41 (0.1–1.19)	0.09	0.98
DQB1	03:02	0.09	0.13	1.47 (0.61–3.31)	0.32	1
DQB1	05:01	0.08	0.05	0.64 (0.16–1.95)	0.42	1
DQB1	05:02	0.07	0.08	1.14 (0.36–3.06)	0.79	1
DQB1	06:01	0.05	0.03	0.48 (0.05–2.12)	0.32	1
DQB1	06:02	0.04	0.07	1.55 (0.42–4.83)	0.41	1
DQB1	06:03	0.05	0.01	0.25 (0.01–1.7)	0.16	1
DQB1	06:04	0.03	0.04	1.5 (0.25–6.44)	0.55	1
DQB1	Binned			0.71 (0.29–1.55)	0.37	1

*p*_c_* = Bonferroni-corrected *p* within DQB1. “binned” pools low-frequency alleles; not interpreted individually.

**Table 4:** HLA-DRB1 allele frequencies and association with ALS.

Locus	Allele	Controls frequency	ALS frequency	OR (95% CI)	*p*-value	*p*_c_*
DRB1	03:01	0.16	0.14	0.89 (0.39–1.86)	0.74	1
DRB1	07:01	0.13	0.32	3 (1.58–5.59)	0.0002	0.001
DRB1	11:04	0.05	0.04	0.73 (0.13–2.65)	0.62	1
DRB1	15:01	0.05	0.09	1.93 (0.64–5.25)	0.16	0.81
DRB1	Binned			0.45 (0.26–0.78)	0.00	0.01

*p*_c_* = Bonferroni-corrected *p* within DRB1. “Binned” pools low-frequency alleles; not interpreted individually.

Under global Bonferroni correction across the 41 non-binned allele-level tests performed at the six classical loci, DQA1*02:01 (raw *p* = 7.23 × 10^-5^; *p*_global_ = 0.003) and DRB1*07:01 (raw *p* = 1.56 × 10^-4^; *p*_global_ = 0.006) remained significant. DQB1*02:02 (raw *p* = 2.53 × 10^-3^; *p*_global_ = 0.10) did not remain significant after across-locus correction. The extended class II haplotype DQA1*02:01~DQB1*02:02~DRB1*07:01 remained significant under global correction across the haplotypes tested at the class II set-locus (*p*_global_ = 0.002).

### Stepwise logistic regression analysis of the class II signal

We next performed stepwise logistic regression to determine whether the three associated class II alleles reflected independent effects or a shared haplotypic signal (**Supplementary Table 4**). Among the 38 ALS cases, DQA1*02:01 and DRB1*07:01 showed complete concordance, with 21 dual carriers and no discordant individuals. Among the 150 controls, only 6 individuals (4.0%) carried one allele without the other. DQB1*02:02 showed greater decoupling from this pair. In univariate models, all three alleles were associated with ALS: DQA1*02:01 (OR = 3.77, 95% CI: 1.80–7.90, *p* = 4 × 10^-4^), DRB1*07:01 (OR = 3.52, 95% CI: 1.68–7.34, *p* = 8 × 10^-4^), and DQB1*02:02 (OR = 2.56, 95% CI: 1.23–5.34, *p* = 0.012). After conditioning on DQA1*02:01 or DRB1*07:01, the DQB1*02:02 association was attenuated to non-significance (OR = 0.74, 95% CI: 0.21–2.53, *p* = 0.63; and OR = 0.85, 95% CI: 0.25–2.84, *p* = 0.79, respectively). Joint models containing both DQA1*02:01 and DRB1*07:01 produced unstable estimates with wide CIs, consistent with near-complete collinearity. A single-predictor model using DR7–DQ2.2 haplotype carrier status yielded OR = 3.24 (95% CI: 1.52–6.88, *p* = 0.002), and the three-allele joint model did not improve fit over the single-haplotype model (likelihood-ratio test: χ² = 3.75, df = 2, *p* = 0.15). Together, these findings indicate that the observed class II association is best interpreted as a single haplotypic signal centred on DR7–DQ2.2 rather than three independent allele-level effects.

### HLA haplotype associations

No class I haplotype met the corrected significance threshold (**Supplementary Table 5**).

The class II haplotype DQA1*02:01~DQB1*02:02~DRB1*07:01 (DR7–DQ2.2) was significantly enriched in ALS cases (OR = 3.11, 95% CI: 1.53–6.19, *p*_c_ = 0.002; **Table 5**). No other class II or combined class I–class II haplotype survived correction (**Supplementary Table 6**).

**Table 5:** Comparison of DRB1~DQA1~DQB1 haplotype frequencies between individuals with ALS and controls.

DQA1~DQB1~DRB1	Controls frequency	ALS frequency	OR (95% CI)	*p*-value	*p*_c_*
01:03~06:03~13:01	0.04	0.01	0.29 (0.01–2.02)	0.21	1
02:01~02:02~07:01	0.1	0.25	3.11 (1.53–6.19)	0.0003	0.002
05:01~02:01~03:01	0.15	0.14	0.98 (0.43–2.07)	0.97	1
05:05~03:01~11:04	0.05	0.04	0.84 (0.15–3.12)	0.79	1
Binned			0.62 (0.36–1.07)	0.06	0.32

*p*_c_* = Bonferroni-corrected *p* across haplotypes within the HLA-DQA1~DQB1~DRB1 set-locus. “Binned” pools low-frequency haplotypes; not interpreted individually.

### HLA amino-acid residue associations

No class I residue reached significance after correction (**Supplementary Table 7**).

Within class II, omnibus tests identified significant differences at DQA1 positions 47, 52, and 54 and DRB1 positions 11, 13, 14, 25, and 30 (**Table 6**). Risk-associated residues in DQA1 (Lys47, His52, and Leu54; each 0.32 in cases vs. 0.13 in controls; OR = 3.18, *p*_c_* = 0.006) and DRB1 (Gly11, Tyr13, Lys14, Gln25, and Leu30; OR = 2.84, *p*_c_* = 0.03) were enriched in cases. Complementary residues at the same positions were depleted (e.g., DQA1-Phe54 OR = 0.31; DRB1-Glu14/Arg25 OR = 0.35). No DQB1 residue survived correction.

**Table 6:** HLA class II amino-acid residues associated with ALS after multiple-testing correction.

Locus	Position	Residue	Controls frequency	ALS frequency	OR (95% CI)	*p*-value	*p*_c_*
DQA1	Pos.47	K	0.13	0.32	3.18 (1.67–5.95)	7.23E−05	0.006
DQA1	Pos.52	H	0.13	0.32	3.18 (1.67–5.95)	7.23E−05	0.006
DQA1	Pos.54	F	0.87	0.68	0.31 (0.17–0.60)	7.23E−05	0.006
DQA1	Pos.54	L	0.13	0.32	3.18 (1.67–5.95)	7.23E−05	0.006
DRB1	Pos.11	G	0.14	0.32	2.84 (1.50–5.26)	0.0003	0.03
DRB1	Pos.13	Y	0.14	0.32	2.84 (1.50–5.26)	0.0003	0.03
DRB1	Pos.14	E	0.86	0.68	0.35 (0.19–0.67)	0.0003	0.03
DRB1	Pos.14	K	0.14	0.32	2.84 (1.50–5.26)	0.0003	0.03
DRB1	Pos.25	Q	0.14	0.32	2.84 (1.50–5.26)	0.0003	0.03
DRB1	Pos.25	R	0.86	0.68	0.35 (0.19–0.67)	0.0003	0.03
DRB1	Pos.30	L	0.14	0.32	2.84 (1.50–5.26)	0.0003	0.03

*p*_c_* = Bonferroni-corrected *p* within each HLA gene (locus-specific).

These residues correspond to the amino-acid signatures that define DQA1*02:01 and DRB1*07:01, indicating that the allele-level and residue-level signals are convergent rather than independent.

### HLA amino-acid associations and structural modelling

**Figure 1:**
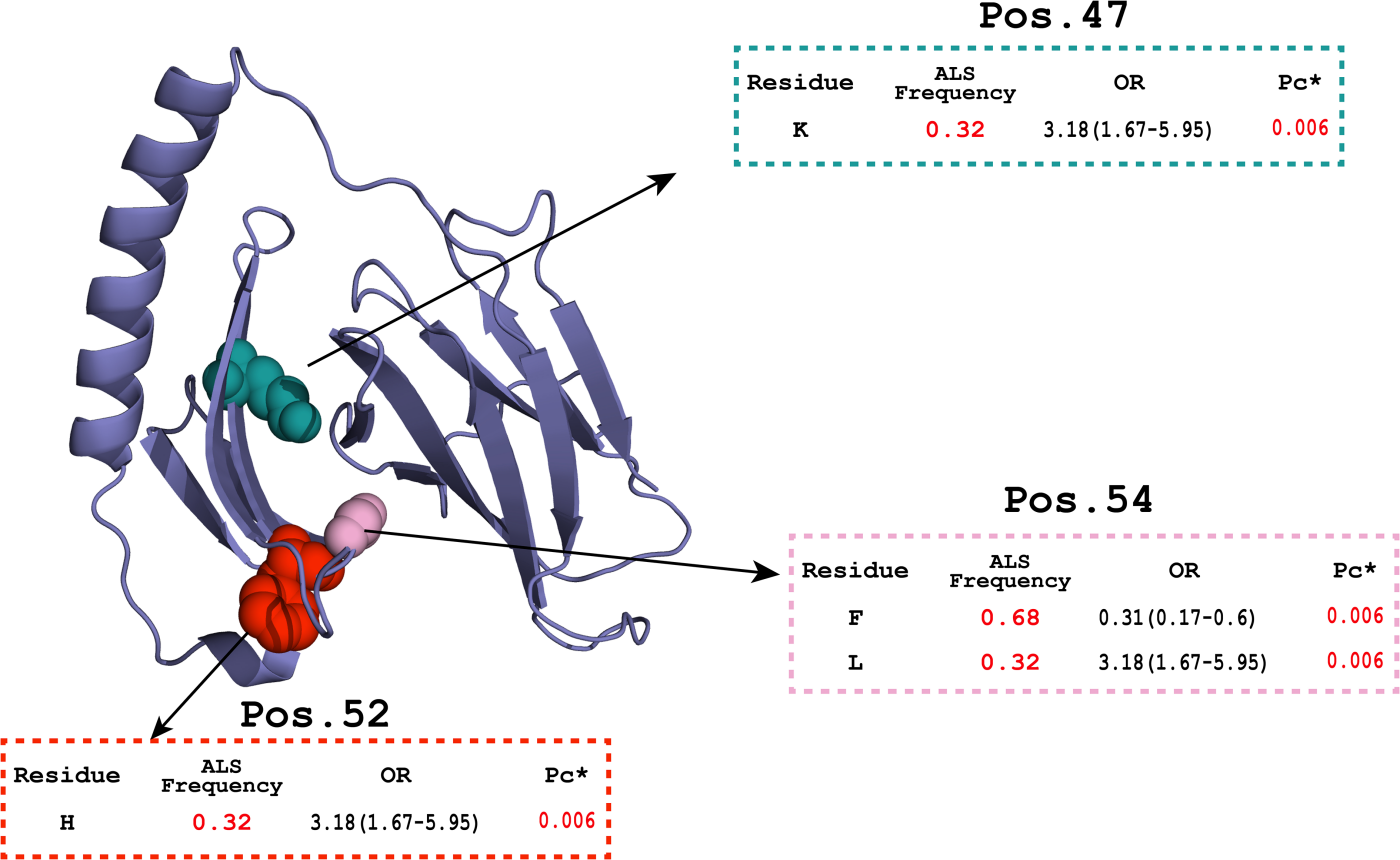
AlphaFold 3 model of the HLA-DQA1*02:01 α-chain highlighting ALS-associated polymorphic residues in the α1 domain.

**Figure 2:**
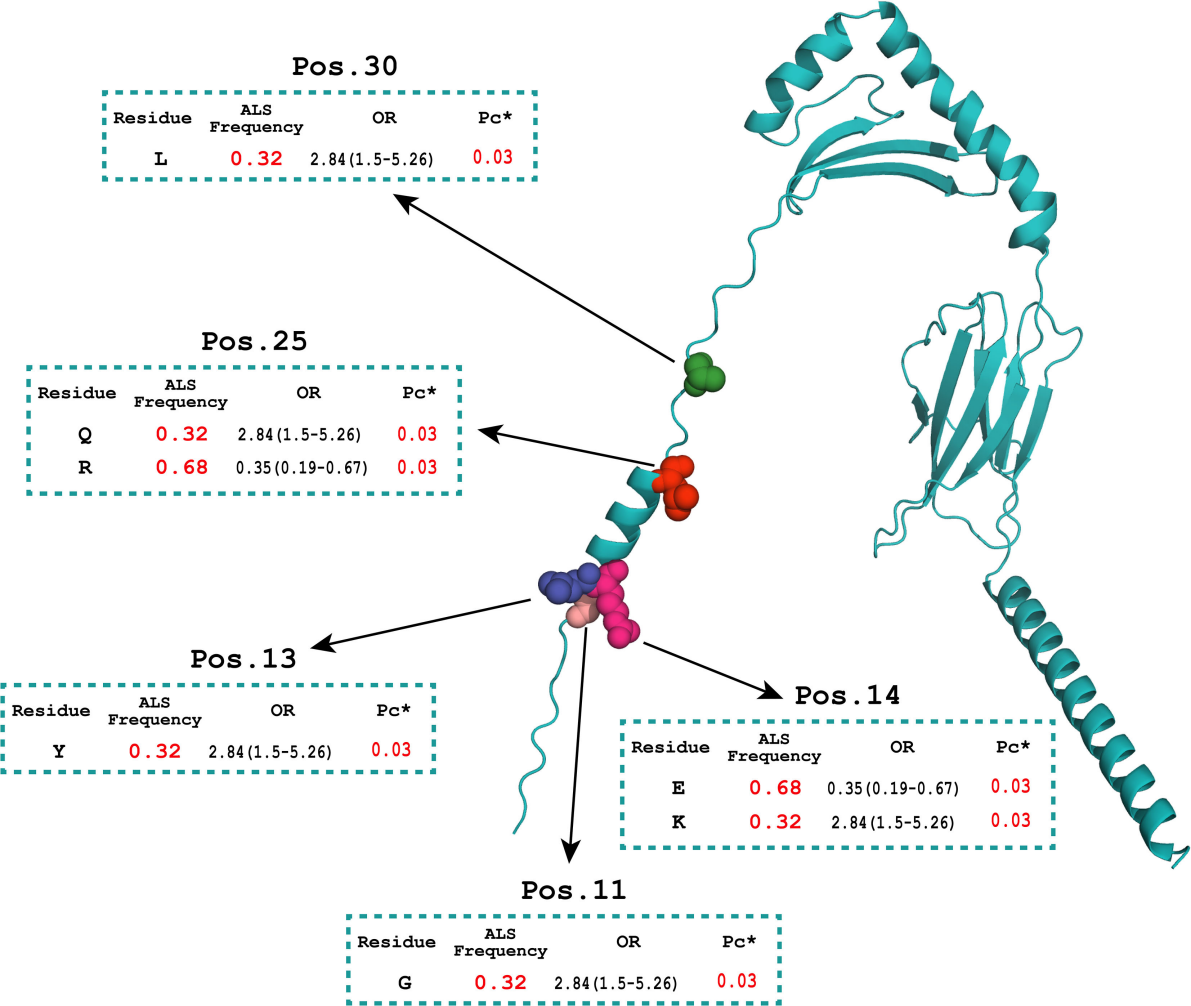
AlphaFold 3 model of the HLA-DRB1*07:01 β-chain highlighting ALS-associated polymorphic residues in the β1 domain.

AlphaFold 3 models of the HLA-DQA1*02:01 α-chain and HLA-DRB1*07:01 β-chain were generated to represent the ALS-associated amino-acid positions in structural context (**Figures 1**, **2**). In DQA1, positions 52 and 54 map to the α1 helix that forms a wall of the peptide-binding cleft in the full DQ αβ heterodimer; α52 lies within the polymorphic α44–54 segment that lines the P1 pocket and contributes to the αβ dimerisation interface (44). Position 47 lies in the adjacent β-sheet floor and may influence local conformation or αβ pairing (**Figure 1**). In DRB1, positions 11–14 and 30 localise to the β-sheet floor/pocket region of the β1 domain, with β11 contributing to pocket 6 and β13 contributing to pocket 4 (42, 45); position 25 lies in an adjacent loop/β-sheet-floor region rather than on the β1 helix (**Figure 2**). Because these are single-chain models, pocket-level interpretations should be confirmed using heterodimeric class II crystal structures.

Coloured spheres indicate positions 47, 52, and 54 in the α1 domain. Positions 52 and 54 map to the α1 helix that contributes to the wall of the peptide-binding cleft in the DQ αβ heterodimer; α52 lies within the polymorphic α44–54 segment that lines the P1 pocket and participates in the αβ dimerisation interface (44). Position 47 lies in the β-sheet floor and may influence local conformation or αβ pairing. Interpretation is limited by the absence of the DQ β-chain in this single-chain model.

Coloured spheres mark positions 11, 13, 14, 25, and 30. Positions 11–14 and 30 localise to the β-sheet floor/pocket region, with β11 contributing to pocket 6 and β13 to pocket 4 (42, 45). Position 25 is better described as an adjacent loop/β-sheet-floor residue rather than the β1 helix. Together, variation at these sites can alter pocket geometry and the presented peptide repertoire, with downstream consequences for CD4^+^ T-cell recognition.

### Genotype–phenotype analysis

Of the 38 patients with ALS, 28 (73.7%) had complete data for age-of-onset analysis. The mean age of onset was 54.7 ± 13.2 years. Among these, 12 carried the DRB1*07:01~DQA1*02:01~DQB1*02:02 risk haplotype (mean onset: 55.0 ± 14.6 years) and 16 were non-carriers (mean onset: 54.4 ± 12.5 years). In the linear regression model including individual alleles (DRB1*07:01, DQA1*02:01, and DQB1*02:02) and adjusting for sex and site of onset, no allele was significantly associated with age of onset (all *p* > 0.25; *R*² = 0.076, *F*-test *p* = 0.87; **Supplementary Figure 1A**). Similarly, the composite risk haplotype model showed no significant association (β = 0.31 years, 95% CI: −10.69 to 11.31, *p* = 0.95). Mann–Whitney *U* tests confirmed no significant difference in age of onset between carriers and non-carriers for any individual allele or the risk haplotype (all *p* > 0.50; **Supplementary Figure 2**).

For survival analysis, 12 patients had documented respiratory end points: four tracheostomies and two NIV >23 h daily (six events total), with 6 patients censored (no respiratory support). Among the 12 patients, 5 carried the risk haplotype (three events) and 7 were non-carriers (three events). In univariate Cox proportional hazards regression, the risk haplotype was not significantly associated with time to respiratory end point (HR = 0.33, 95% CI: 0.03–3.39, *p* = 0.35; **Supplementary Figure 1B**). DRB1*07:01 showed a non-significant trend towards a protective effect (HR = 0.13, 95% CI: 0.01–1.41, *p* = 0.09). After adjustment for age of onset, the risk haplotype remained non-significant (HR = 0.27, 95% CI: 0.02–3.81, *p* = 0.34). Kaplan–Meier analysis showed no significant difference in event-free survival between risk haplotype carriers and non-carriers (log-rank *p* = 0.33; **Supplementary Figure 3**).

## Discussion

We tested whether variation in classical HLA class I (HLA-A, -B, and -C) and class II (HLA-DRB1, -DQA1, and -DQB1) is associated with ALS in a Kuwaiti case–control cohort using HLA genotypes derived from NGS. We performed allele, haplotype, and amino-acid residue association analyses, followed by exploratory genotype–phenotype testing within cases. For HLA class I, we did not detect associations that remained significant after multiple-testing correction at any level. This differs from early serology-based candidate studies that reported signals for HLA-B35 and protective effects for HLA-A9 (46–50), but those reports were limited by low typing resolution and small sample sizes. A recent meta-analysis of eight studies suggests that class I effects in ALS are modest and difficult to replicate across ancestries (29), which is consistent with our null results.

For HLA class II, we observed a consistent risk signal centred on HLA-DQA1*02:01, HLA-DQB1*02:02, and HLA-DRB1*07:01, and on the extended haplotype DQA1*02:01~DQB1*02:02~DRB1*07:01 (DR7–DQ2.2), which conferred approximately a threefold higher odds of ALS. Amino-acid analysis provided additional resolution: DQA1 positions 47 (Lys), 52 (His), and 54 (Leu) in the α1 domain were present in approximately 32% of cases compared with 13% of controls, and DRB1 positions 11 (Gly), 13 (Tyr), 14 (Lys), 25 (Gln), and 30 (Leu) were similarly enriched. At the same time, Glu14 and Arg25 were depleted in the patient group (OR range = 2.8 to 3.2 for risk residues). Alternative residues at the same positions showed protective trends, providing functional resolution that goes beyond allele-level associations (45, 51). These residues match the defining amino-acid motifs of DQA1*02:01 and DRB1*07:01, indicating that the residue-level and allele-level results are largely driven by the same underlying haplotype rather than independent effects. To interpret these residues structurally, we mapped them onto AlphaFold 3 single-chain models and considered them alongside published class II crystal structures (42, 43). In DQA1, positions 52 and 54 lie on the α1 helix that forms a wall of the peptide-binding cleft in the intact DQ αβ heterodimer, and α52 falls within the polymorphic α44–54 segment implicated in P1 pocket lining and the αβ dimerisation interface (44, 52). Position 47 lies in the β-sheet floor and may influence local conformation or αβ pairing. In DRB1, positions 11 to 14 and 30 localise to the peptide-binding site and pocket floor region of the β1 domain, with β11 contributing to pocket 6 and β13 to pocket 4, whereas position 25 is better described as an adjacent loop or β-sheet-floor residue (45). Variation at these sites can alter pocket geometry and thereby the repertoire of peptides presented to CD4^+^ T cells, a mechanism supported by residue-level mapping in other immune-mediated diseases. In rheumatoid arthritis, DRB1 positions 11/13/71/74 explain the majority of MHC-associated risk through pocket-level alterations (45), and in type 1 diabetes, specific DQ/DR residue configurations modulate autoantigen binding affinity and disease susceptibility (53–55). No independent DQB1 residues reached significance, which is expected given the context dependency of the DQ heterodimer. Trans-encoded dimers in heterozygous individuals may amplify effects, although rare cross-DR/DQ pairings are unlikely to occur at appreciable frequency (56, 57). Our data therefore suggest that this DQα and DRβ residue motif may alter the spectrum of peptides presented in ALS-relevant immune responses, supporting a contribution of class II-restricted antigen presentation to neuroinflammation. Because our models are single chains, pocket-level assignments should ultimately be confirmed using heterodimeric structures and functional binding assays to determine which specific positions are mechanistically causal.

Stepwise logistic regression of the three class II alleles supported a single underlying haplotypic effect rather than three independent signals. DQB1*02:02 lost its association after conditioning on either DQA1*02:01 or DRB1*07:01, and the three-allele joint model did not fit better than the single-haplotype model (likelihood-ratio *p* = 0.15). In our cohort, DQA1*02:01 and DRB1*07:01 showed near-complete collinearity, limiting our ability to distinguish their individual contributions statistically. Distinguishing the individual causal contribution of either allele, or determining whether risk is mediated jointly through the assembled class II heterodimer, will require larger cohorts containing informative recombinant haplotypes and direct functional peptide-binding studies.

The DR7–DQ2.2 haplotype has been implicated in other immune-related phenotypes. It increases the risk of asparaginase hypersensitivity in paediatric acute lymphoblastic leukaemia, with carriers showing fivefold higher odds, probably through enhanced presentation of drug-derived peptides (58). DRB1*07:01 and DQA1*02:01 have also been associated with anti-LGI1 encephalitis, an autoimmune neurological condition (59). In the broader ALS-HLA literature, class II markers such as DR4 (selected HLA-DRB1*04 alleles) have reached only nominal significance and did not survive correction for multiple testing (29), suggesting modest aggregate effects but still pointing towards HLA-mediated immunity. Evidence from other ancestries supports class II involvement: in Han Chinese, a DR/DQ-region variant (rs9268856) associates with both increased ALS susceptibility and shorter survival (32). A class II signal is also compatible with recent immunological findings. Patients with ALS mount CD4^+^ T-cell responses against *C9orf72*, with responses skewed towards *IL-5*/*IL-10* and higher *IL-10* associated with longer predicted survival. Mapped epitopes span the C9orf72 protein with HLA-II restrictions to DRB1*14:01, DRB1*03:01, DRB1*01:02, and DQB1*05:01, and predicted promiscuous binding capacity across common class II alleles (60). The class II signals we observed and their magnitude are consistent with population-specific HLA architecture and gene–environment interplay. Deployment during the 1991 Gulf War was associated with approximately a twofold higher risk of ALS (5), illustrating the strong impact of regional non-genetic factors. Our results, therefore, strengthen the rationale for studying class II-restricted antigen presentation and neuroinflammation in ALS in a population-specific context, shaped by local allele frequencies, linkage disequilibrium patterns, and regional exposures. Translationally, these findings also suggest that antigen presentation rather than cytotoxic immunity may represent the dominant HLA-mediated mechanism in ALS susceptibility.

We also explored whether the class II risk alleles or the composite DR7–DQ2.2 haplotype influenced age of onset or time to respiratory end point. We did not detect significant associations, but this analysis was underpowered: only 28 cases had age-of-onset data and only 12 had end point documentation with 6 events, well below the recommended 10 events per covariate for reliable Cox regression (Peduzzi et al., 1996). Larger cohorts with systematic longitudinal follow-up will be required to assess whether HLA variation contributes to disease course in addition to susceptibility.

This study has limitations. The sample size was modest for a rare disease, which reduces power for low-frequency alleles and residue variants and increases uncertainty in effect-size estimates. This constraint is difficult to avoid given the low global incidence of ALS (4) and the small base population of Kuwait. However, the consistency across allele-, haplotype-, and residue-level analyses substantially reduces the likelihood of a false-positive signal despite modest sample size.

The sex imbalance between cases and controls may introduce residual confounding, although HLA is autosomal (6p21) and any sex-related influence would therefore be indirect. Cases and controls were sequenced on different platforms (WGS in cases and WES in controls). We mitigated this by re-typing both cohorts with HLA-HD and analysing genotypes at two-field resolution, where our prior evaluation showed high cross-platform concordance (94.6% at the second field and >95% overall across 29 HLA loci; 34), but subtle platform effects cannot be fully excluded. The genotype–phenotype analyses were constrained by the small number of patients with complete clinical and end point data (*n* = 12 for the survival analysis), so we cannot draw firm conclusions about the effects of HLA on disease progression. We also lacked an independent regional replication cohort. Validation in neighbouring Gulf populations, together with functional work such as peptide-binding assays and heterodimer modelling with bound peptides, will be important for confirming the association and defining the causal residue(s). Given that our observational genetic study lacks direct functional validation, our mechanistic interpretations of the residue-level findings should be viewed as provisional.

## Conclusion

Using high-resolution NGS-based HLA typing in a Kuwaiti ALS case–control cohort, we identified a class II risk signal centred on the DRB1*07:01~DQA1*02:01~DQB1*02:02 (DR7–DQ2.2) haplotype, which conferred approximately threefold higher odds of ALS, with convergent amino-acid residue signals in the DQα and DRβ peptide-binding domains. These findings support a contribution of class II-restricted antigen presentation to ALS susceptibility and warrant functional validation and replication in larger, independent Middle Eastern cohorts.

## Data Availability

The datasets presented in this study can be found in online repositories. The names of the repository/repositories and accession number(s) can be found in the article/**Supplementary Material**.

## Supplementary material

Supplementary Figure 1 Forest plot of genotype–phenotype associations. **(A)** Linear regression coefficients (β, in years) for the association between the DRB1*07:01~DQA1*02:01~DQB1*02:02 risk haplotype, male sex, and bulbar onset with age of ALS onset (n = 28). The dashed line at zero indicates no effect. **(B)** Univariate Cox proportional hazards regression showing hazard ratios for time to respiratory end point (tracheostomy or NIV >23 hours daily) by risk haplotype, DRB1*07:01 carrier status, male sex, and bulbar onset (n = 12, 6 events). The dashed line at HR = 1 indicates no effect. Error bars represent 95% confidence intervals. No associations reached statistical significance.

Supplementary Figure 2 Age of ALS onset by HLA risk allele carrier status. Box plots show the distribution of age of onset for carriers (red) versus non-carriers (blue) of **(A)** DRB1*07:01, **(B)** DQA1*02:01, **(C)** DQB1*02:02, and **(D)** the composite DRB1*07:01~DQA1*02:01~DQB1*02:02 risk haplotype. Individual data points are overlaid. Mann–Whitney U test P-values are shown above each comparison. No significant differences were observed (n = 28).

Supplementary Figure 3 Kaplan–Meier event-free survival by HLA risk haplotype status. Survival curves compare time from symptom onset to respiratory end point (tracheostomy or NIV >23 hours daily) between carriers (red; n = 5) and non-carriers (blue; n = 7) of the DRB1*07:01~DQA1*02:01~DQB1*02:02 risk haplotype. Vertical tick marks indicate censored observations. Shaded areas represent 95% confidence intervals. The number at risk is shown below the plot. Log-rank test P = 0.33.
