# Supplementary figures and images for: Classical HLA class II associations with ALS in Kuwait reveal a DR7–DQ2.2 risk haplotype
Source: Front Immunol. 2026 May 8;17:1820694. doi: 10.3389/fimmu.2026.1820694 (PMC13194005; doi:10.3389/fimmu.2026.1820694)

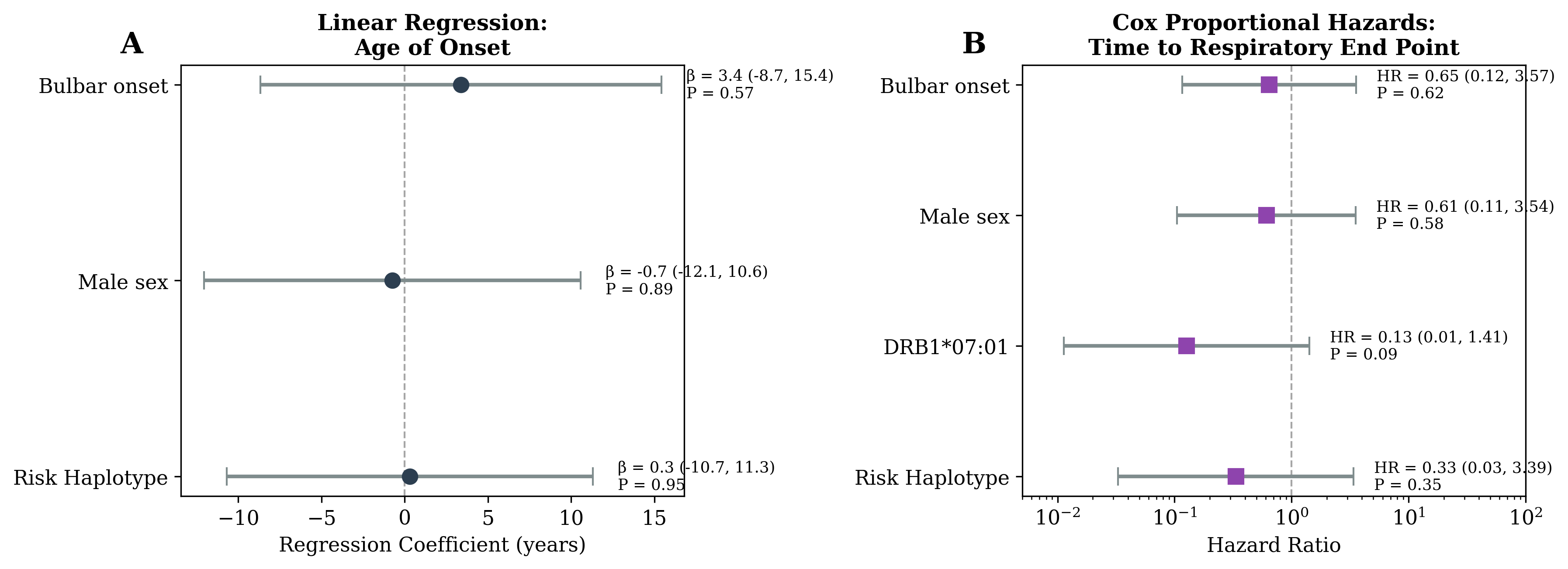

Supplement: Supplementary Figure 1 — Forest plot of genotype–phenotype associations. (A) Linear regression coefficients (β, in years) for the association between the DRB1*07:01~DQA1*02:01~DQB1*02:02 risk haplotype, male sex, and bulbar onset with age of ALS onset (n = 28). The dashed line at zero indicates no effect. (B) Univariate Cox proportional hazards regression showing hazard ratios for time to respiratory end point (tracheostomy or NIV >23 hours daily) by risk haplotype, DRB1*07:01 carrier status, male sex, and bulbar onset (n = 12, 6 events). The dashed line at HR = 1 indicates no effect. Error bars represent 95% confidence intervals. No associations reached statistical significance. [file Image1.png]

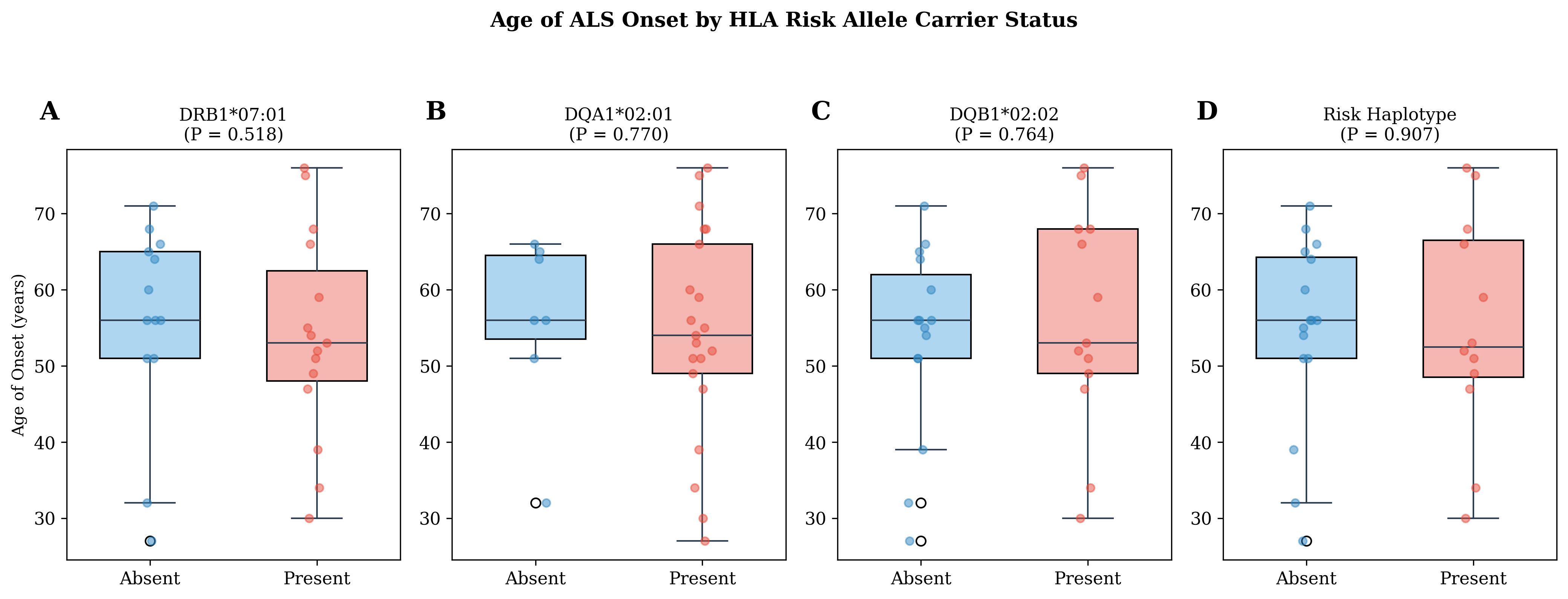

Supplement: Supplementary Figure 2 — Age of ALS onset by HLA risk allele carrier status. Box plots show the distribution of age of onset for carriers (red) versus non-carriers (blue) of (A) DRB1*07:01, (B) DQA1*02:01, (C) DQB1*02:02, and (D) the composite DRB1*07:01~DQA1*02:01~DQB1*02:02 risk haplotype. Individual data points are overlaid. Mann–Whitney U test P-values are shown above each comparison. No significant differences were observed (n = 28). [file Image2.png]

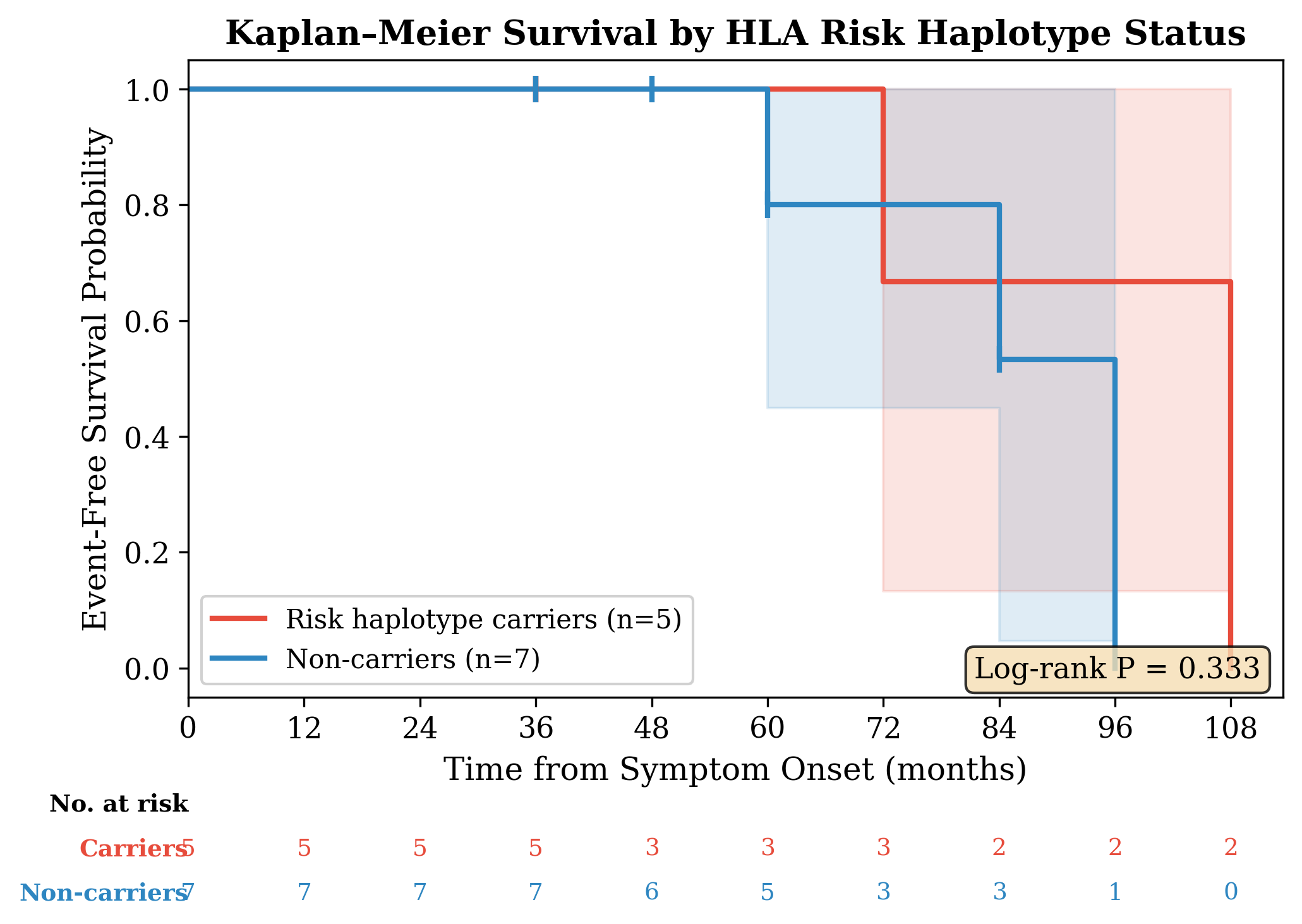

Supplement: Supplementary Figure 3 — Kaplan–Meier event-free survival by HLA risk haplotype status. Survival curves compare time from symptom onset to respiratory end point (tracheostomy or NIV >23 hours daily) between carriers (red; n = 5) and non-carriers (blue; n = 7) of the DRB1*07:01~DQA1*02:01~DQB1*02:02 risk haplotype. Vertical tick marks indicate censored observations. Shaded areas represent 95% confidence intervals. The number at risk is shown below the plot. Log-rank test P = 0.33. [file Image3.png]
